# Flow field characteristics and experimental research on inner-jet electrochemical face grinding of SUS420J2 stainless steel

**DOI:** 10.1038/s41598-022-16099-1

**Published:** 2022-07-11

**Authors:** Feng Wang, Yafeng He, Xiaokai Wu, Min Kang

**Affiliations:** 1 College of Engineering, Nanjing Agricultural University, Nanjing, 210031 China; 2 Jiangsu Key Laboratory of Precision and Micro-Manufacturing Technology, Nanjing University of Aeronautics and Astronautics, Nanjing, 210016 China; 3 School of Aeronautical and Mechanical Engineering, Changzhou Institute of Technology, Changzhou, 213032 China

**Keywords:** Mechanical engineering, Materials science

## Abstract

Electrochemical grinding (ECG) is processed by the combination of dissolution and grinding. It is very suitable for the processing of difficult-to-cut stainless steel, but its processing performance is restricted by the matching effect of dissolution and grinding. In this work, the processing of the torus surfaces of the stainless steel shaver cap was taken as the research object. A flow field model including the through-hole structure and the rotation of the grinding head was proposed to optimize the flow field distribution and promote the uniform dissolution of materials. The flow field simulation results showed that the rotational flow formed by the high-speed rotation prolonged the electrolyte flow path and was not conducive to the discharge of electrolytic products, and the reasonable selection of the diameter and distribution of the through-hole could reduce the velocity difference. The effects of rotational speed, feed rate, and inlet pressure on the flatness and surface roughness of the torus surfaces were experimentally investigated, and a better matching effect of dissolution and grinding was obtained. Moreover, the experimental results showed that the inner-jet ECG had a good prospect in the batch processing of high-hardness stainless steel parts.

## Introduction

SUS420J2 stainless steel has excellent corrosion resistance, high strength, and wear resistance after heat treatment, and has a wide range of applications in valve seats, and cutting tools. However, as the hardness of stainless steel increases, the traditional cutting is prone to severe tool wear. Electrochemical grinding (ECG) is a combination of dissolution and grinding to achieve material removal, and it has significant advantages in the processing of difficult-to-cut materials^1,2^. Traditional ECG usually uses conductive grinding heads with abrasive grains for material removal^3^. In recent years, to further improve the processing quality, methods such as ultrasonic-assisted ECG^4,5^, electrochemical jet-assisted grinding^6^, dry electrochemical mechanical machining^7^, and abrasive-free electrochemical mechanical machining^8^ have been developed, and the application of ECG in the processing of metals and semiconductor materials has been promoted by these methods. In addition, many scholars have tried to promote the matching effect of dissolution and grinding by improving the flow field distribution.

Previous studies have shown that the tool structure and movement mode have important effects on the flow field distribution and material removal^9–13^. In terms of the tool movement, Mitchell-Smith et al.^14^ improved the electrochemical jet machining performance of Inconel 718 alloy by adjusting the jet angle through nozzle rotation. Mishra et al.^15^ used the tool rotation to improve the flow field distribution of Nimonic-263 alloy electrochemical milling, and eliminated the flow disorder on the bottom surface. Zhao et al.^16^ used the tool vibration to eliminate the sudden change of flow field in the frontal gap of special-shaped holes electrochemical machining (ECM), and promoted the uniform dissolution of stainless steel materials. In terms of the tool structure, Pang et al.^17^ used a floating cathode to process the outer surface of GCr15 steel rotating parts, and established the relationship between the inter-electrode gap and the electrolyte pressure. Li et al.^18^ analyzed the fluid flow structure of the tubular grinding wheel based on the flow field simulation, and showed that the spiral fluid flow groove could improve the uniformity of the flow velocity in the lateral gap. In addition, the influence of different inner-jet grinding wheel structures on the flatness and the frontal gap distribution of the GH4169 alloy ECG was also studied^19^. Yue et al.^20^ optimized the flow velocity distribution in the frontal gap by opening through-holes at the bottom of the grinding head, thereby improving the flatness of the bottom surface of Inconel 718 alloy electrochemical milling-grinding. In the above research, the dissolution of the materials was effectively improved by optimizing the flow structure and movement rule of the tool. However, the research objects were concentrated on the ECM of the holes and grooves, and less attention was paid to the electrochemical face grinding (ECFG) of difficult-to-cut materials.

In this work, the inner-jet ECFG of the heat-treated SUS420J2 stainless steel shaver cap was investigated. A flow field model including the through-holes on the sidewall of the grinding head and the rotation of the grinding head was proposed. The influence of the rotation speed, the diameter and distribution of the through-hole on the flow field fluctuations in the inner and outer torus surface of the shaver cap was studied based on the flow field simulation. In addition, the influences of electrolyte pressure, rotation speed, and feed rate on the flatness and surface roughness of the torus surfaces were analyzed based on experimental research. The optimized matching effect of dissolution and grinding was promoted, and the ECG of SUS420J2 stainless steel was improved.

## Principle of inner-jet ECFG

**Figure 1:**
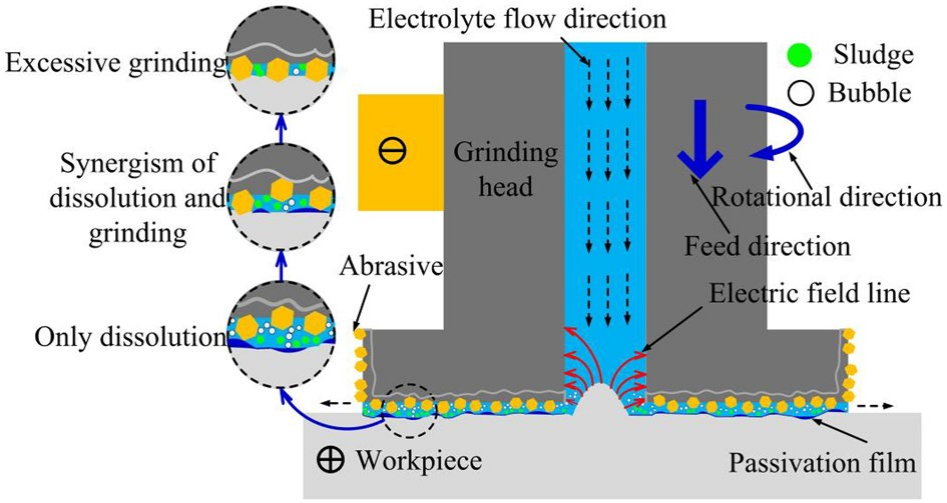
Principle of inner-jet ECFG.

The principle of inner-jet ECFG is depicted in Fig. 1. The conductive grinding head with abrasive grains rotates around the axis and feeds towards the workpiece at the same time, the high-speed rotating grinding head is connected to the negative pole of the power supply and the workpiece is connected to the positive pole. The high-velocity electrolyte flows from the inside of the grinding head into the processing area and flows out from the frontal gap. Under the synergistic effect of an external electric field, high-velocity electrolyte, and grinding head movement, the workpiece partially dissolves and covers with the passivation film. The passivation film on the surface protrusions is scraped off by abrasive particles, so that the substrate material is re-dissolved, while the recesses remain passivated. With the alternate dissolution and grinding action, the workpiece surface is processed. However, different processing states will be formed due to the different matching effects of dissolution and grinding, which will affect the pros and cons of ECFG performance. When the frontal gap is large, only the electrochemical reaction occurs on the workpiece surface without abrasive scraping, and the surface macroscopic and microscopic leveling performance is not high. When the frontal gap is small, the abrasive grains cut into the inside of the substrate and form obvious grinding marks, which is also not conducive to the improvement of surface quality.

## Flow field simulation of ECFG

During the dissolution process, the flow field distribution in the inter-electrode gap has an important influence on the electrochemical reaction and the matching effect of dissolution and grinding. To investigate the influence of the grinding head rotation and the through-hole structure on the flow field distribution of the ECG of the shaver cap’s torus surfaces, the analysis was carried out using fluid dynamics simulation.

### Flow field model

**Figure 2:**
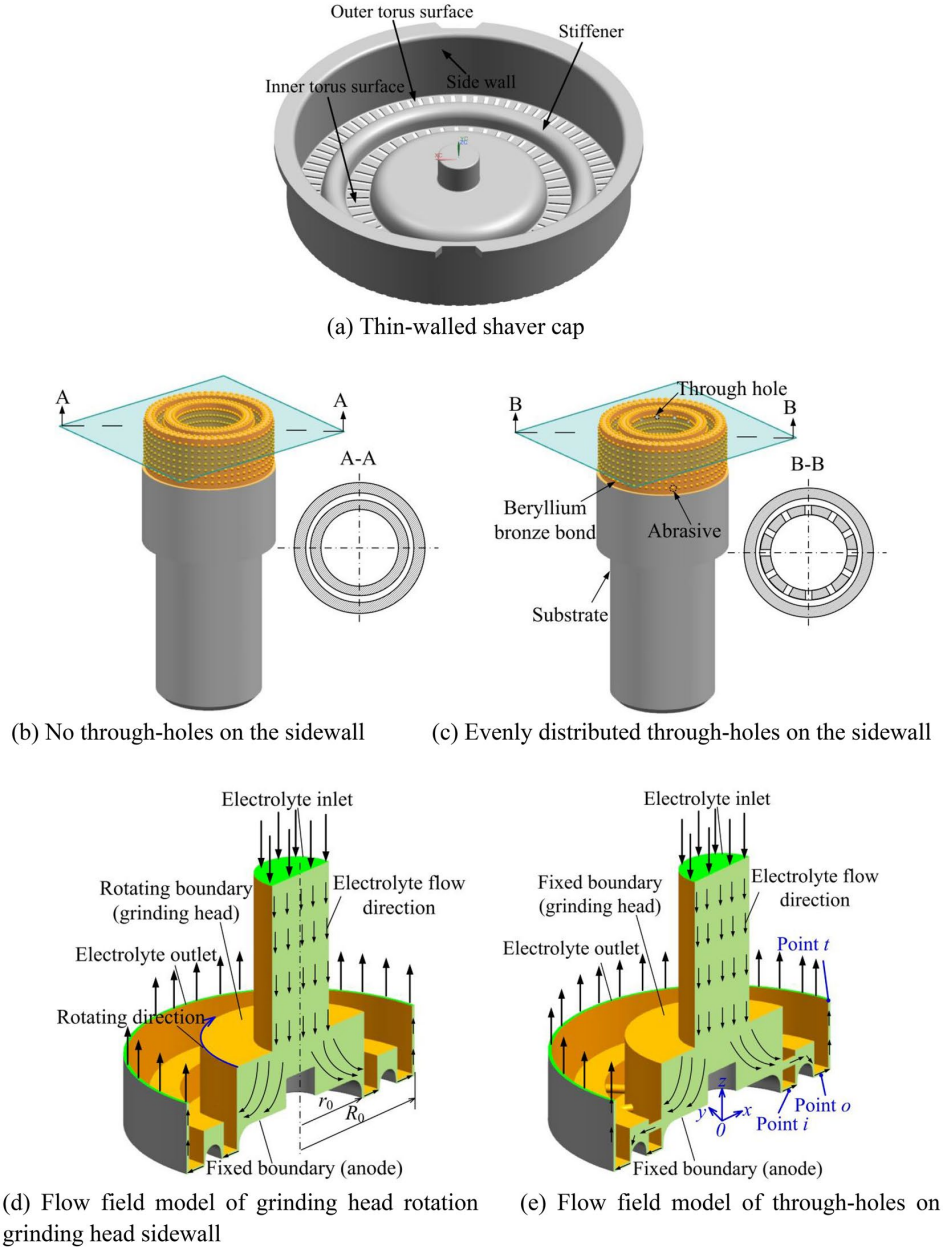
Geometric model of shaver cap, grinding head and flow field.

Figure 2a shows the structure of a thin-walled shaver cap with an outer diameter of 20 mm, and a height of 5 mm. The torus surface to be processed includes an inner torus surface and an outer torus surface, both of which have a width of 1.3 mm and a wall thickness of 0.1 mm. The inner and outer torus surfaces are connected by the stiffener. Figure 2b,c show two different conductive grinding heads. The through-holes on the grinding head sidewall are evenly distributed in the circumferential direction, and the center is 1.5 mm away from the end face of the grinding head. When constructing the flow field model, since the width of the narrow slits evenly distributed on the inner and outer torus surface of the shaver cap is only 0.25 mm, the torus surface is assumed to be a closed plane, and the influence of the group slits on the flow field distribution is ignored. Since the abrasive grain size is less than 100 μm and the exposed height is small, it is assumed that the end surface of the grinding head is a smooth plane. Figure 2d shows the flow field model of the grinding head rotation, *r*_0_ is the radius at the entrance of the frontal gap, and *R*_0_ is the radius at the exit of the frontal gap. The grinding head boundary rotates around the axis, the anode boundary is fixed, and the electrolyte flows in from the hollow area of the grinding head and out from the lateral gap between the grinding head and the workpiece sidewall. Figure 2e shows the flow field model with through-holes on the sidewall of the grinding head. The boundaries of the grinding head and workpiece are fixed. Sampling points *i*, *o,* and *t* correspond to the middle position of the inner torus boundary, outer torus boundary, and electrolyte outlet respectively.

With the aid of turbulence module and rotating machinery module of COMSOL Multiphysics 5.5.0.359 (https://cn.comsol.com/product-download/5.5/windows), the influence of grinding head rotation and through-hole structure on the flow velocity distribution was assessed. The main parameters of flow field simulation are shown in Table 1. Centrifugal force and Coriolis force are introduced to analyze rotation in the rotating machinery model. The Navier–Stokes equations are used to control the momentum balance, and the continuity equation is used to control the conservation of mass in the turbulence model. In addition, the turbulence effect is modeled by the standard *k*-*ε* two-equation model, and the near-wall flow is modeled by the wall function^21^. The turbulence model and the rotating machinery model meet the following assumptions: (1) the electrolyte is an incompressible Newtonian fluid and does not contain hydrogen, oxygen, and solid products; (2) since the length of the electrolyte flow path is less than 5 mm, the influence of temperature rise on fluid flow is ignored^22^; (3) ignoring the effect of the grinding head rotation on the heat transfer, only considering the effect of Coriolis force and centrifugal force on the fluid flow.

**Table 1:** Main parameters of flow field simulation of ECFG.

Simulation parameter	Value
Frontal gap Δ_0_ (mm)	0.1
Rotating speed of grinding head *r* (rpm)	0, 1800, 3000, 4200, 6000, 12,000, 24,000
Number of through-holes on the sidewall of grinding head	0, 3, 6, 9, 12, 15, 18
Diameter of through-hole *d* (mm)	0.4, 0.5, 0.6, 0.7, 0.8
Electrolyte density *ρ* (kg × m^−3^)	1150
Kinematic viscosity coefficient of electrolyte *ν* (m^2^ × s^−1^)	1.01 × 10^–6^
Electrolyte inlet pressure *P*_in_ (MPa)	0.2, 0.25, 0.3, 0.35, 0.4
Electrolyte outlet pressure *P*_out_ (MPa)	0.1

### Influence of grinding head rotation

**Figure 3:**
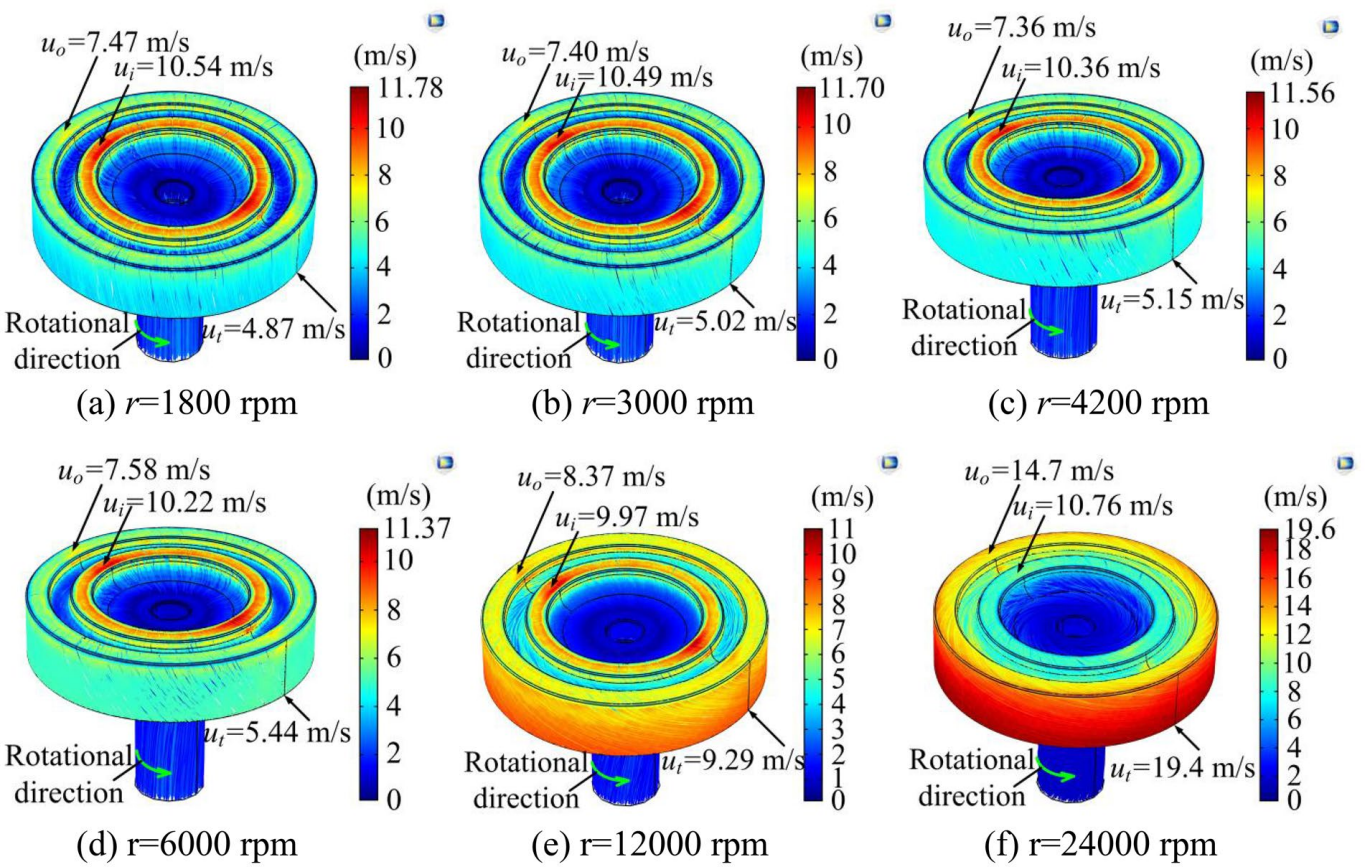
Electrolyte streamline at different rotation speeds.

Figure 3 shows the change of electrolyte streamline at different rotation speeds, the inlet pressure is 0.3 MPa, and the number of through-holes on the sidewall of the grinding head is zero. It can be seen from Fig. 3 that when the rotating speed is lower than 6000 revolutions per minute (rpm), the flow velocity on the inner and outer torus surfaces changes little with the increase of the rotating speed. However, the flow direction near the electrolyte outlet is deflected in the radial direction due to the Coriolis force, and the flow direction deflection increases with the increase in rotating speed. In addition, the flow velocity on the inner torus surface is significantly higher than that on the outer torus surface due to the pressure loss along the flow path and the energy loss caused by the sudden change of the cross-sectional area of the flow channel. The high-velocity electrolyte promotes the dissolution of the inner torus, and resulting in a big difference in the dissolution rate of the inner and outer torus surfaces. When the rotation speed is higher than 12,000 rpm, the rotation of the grinding head has a significant effect on the fluid flow, while the fluctuation of the cross-sectional area of the flow channel has a small effect on the flow velocity. Since the linear speed of the grinding head near the electrolyte outlet is the highest, the flow velocity is also the highest under the action of centrifugal force. In addition, the electrolyte fluid generates a rotating flow under the action of centrifugal force and Coriolis force, and the flow deflection increases significantly with the increase in rotation speed. However, the increased deflection of flow direction significantly prolongs the electrolyte flow path as shown in Fig. 3f, and is not conducive to the timely discharge of electrolytic products in the inter-electrode gap. Moreover, the difference in flow velocity between the inner and outer torus surfaces increases with the increase in rotation speed, which is not conducive to the uniform dissolution of materials.

### Influence of electrolyte pressure

**Figure 4:**
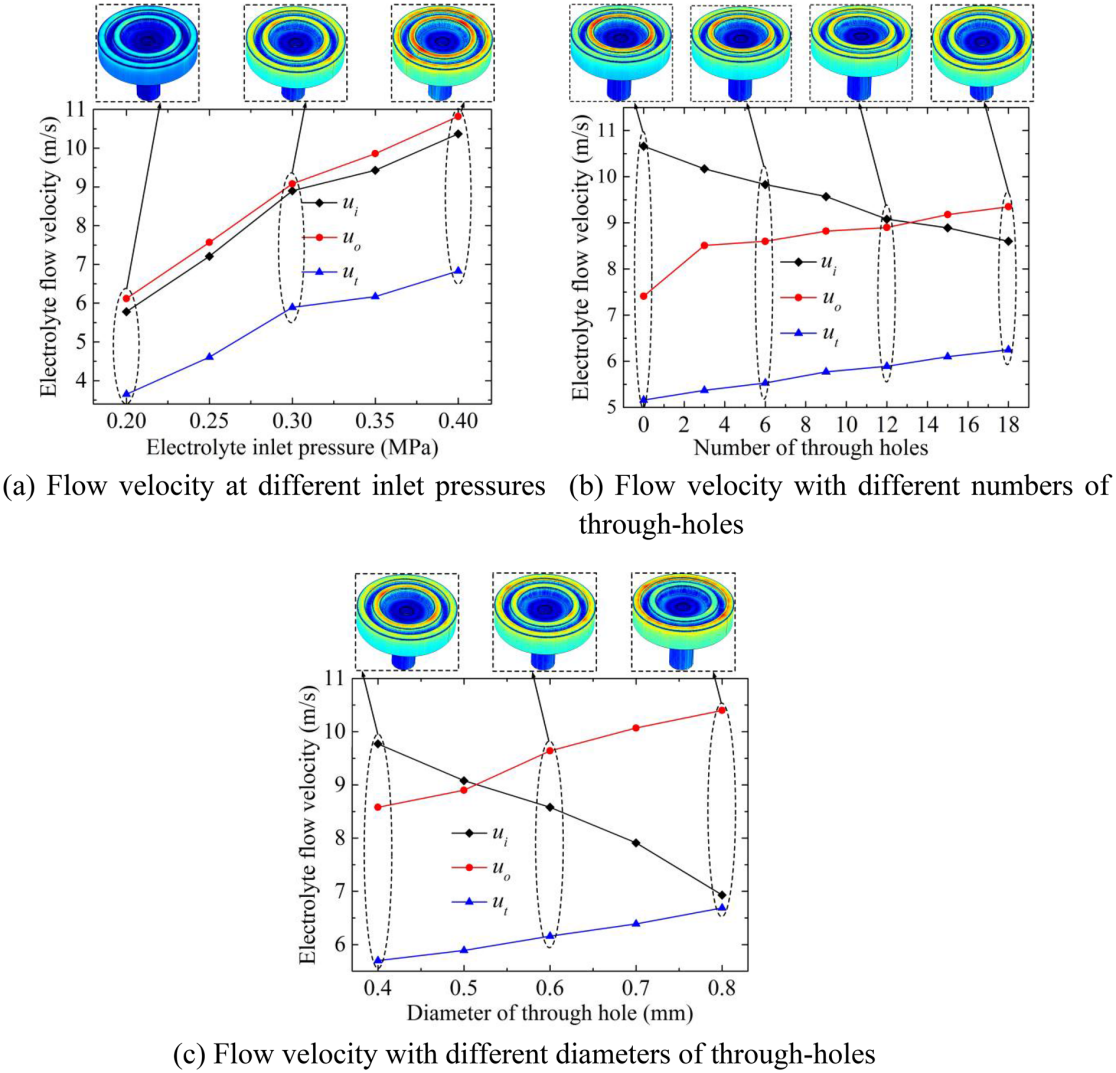
Flow velocity for different inlet pressure and through-hole structure.

Figure 4a shows the change of flow velocity at different inlet pressures. The rotation speed is zero, and the number of through-holes is also zero. It can be seen from Fig. 4a that the conversion of hydrostatic pressure energy increases as the inlet pressure increases, and the flow velocity of the sampling points at the inner and outer torus surface and the electrolyte outlet (*u*_*i*_, *u*_*o*_*, u*_*t*_) increase significantly. In addition, the flow velocity gradient from the inner torus surface to the outlet increases significantly as the inlet pressure increases, which is beneficial to product transportation in the inter-electrode gap, thereby promoting the continuous dissolution process. However, the excessively high inlet pressure causes only the electrochemical reaction on the workpiece surface, which is not conducive to the improvement of the leveling performance of the torus surfaces.

### Influence of number and diameter of through-hole

Figure 4b shows the flow velocity distribution of the different numbers of through-holes on the sidewall of the grinding head. The through-hole diameter is 0.5 mm, and the grinding head rotation speed is zero. Figure 4c shows the flow velocity distribution of different diameters of the through-holes. The number of through-holes is 12, and the rotation speed is also zero. It can be seen from Fig. 4b that with the increase in the number of through-holes, the flow velocities of the sampling points at the inner torus surface are reduced, while the flow velocities of the sampling points at the outer torus surface and the outlet are significantly increased. The difference in flow velocity between the inner and outer torus surfaces is small when the number of through-holes is between 9 and 15, which is beneficial to reduce the fluctuation of the dissolving rate of materials. It can be seen from Fig. 4c that the electrolyte flow in the outer torus surface increases significantly as the diameter of the through-hole increases, resulting in a significant increase in the flow velocity of the sampling points at the outer torus surface and outlet. The flow velocity of the inner and outer torus surfaces is relatively close when the diameter of the through-hole is between 0.4 and 0.6 mm, which is conducive to the uniform dissolution of materials.

Based on the influence of the above-mentioned grinding head rotation and through-hole structure on the flow field distribution, it can be seen that the auxiliary high-speed rotation is not beneficial to the product discharge in the inter-electrode gap, and the large difference in flow velocity between the inner and outer torus surfaces is not conducive to the uniform dissolution of materials. The number and diameter of the through-holes of the grinding head have a significant impact on the flow field. When the through-hole diameter is between 0.4 mm and 0.6 mm and the number of through-holes is between 9 and 15, the flow velocity difference between the inner and outer torus surfaces is small, which is conducive to the optimal matching of dissolution and grinding.

## ECG system and experimental arrangements

**Figure 5:**
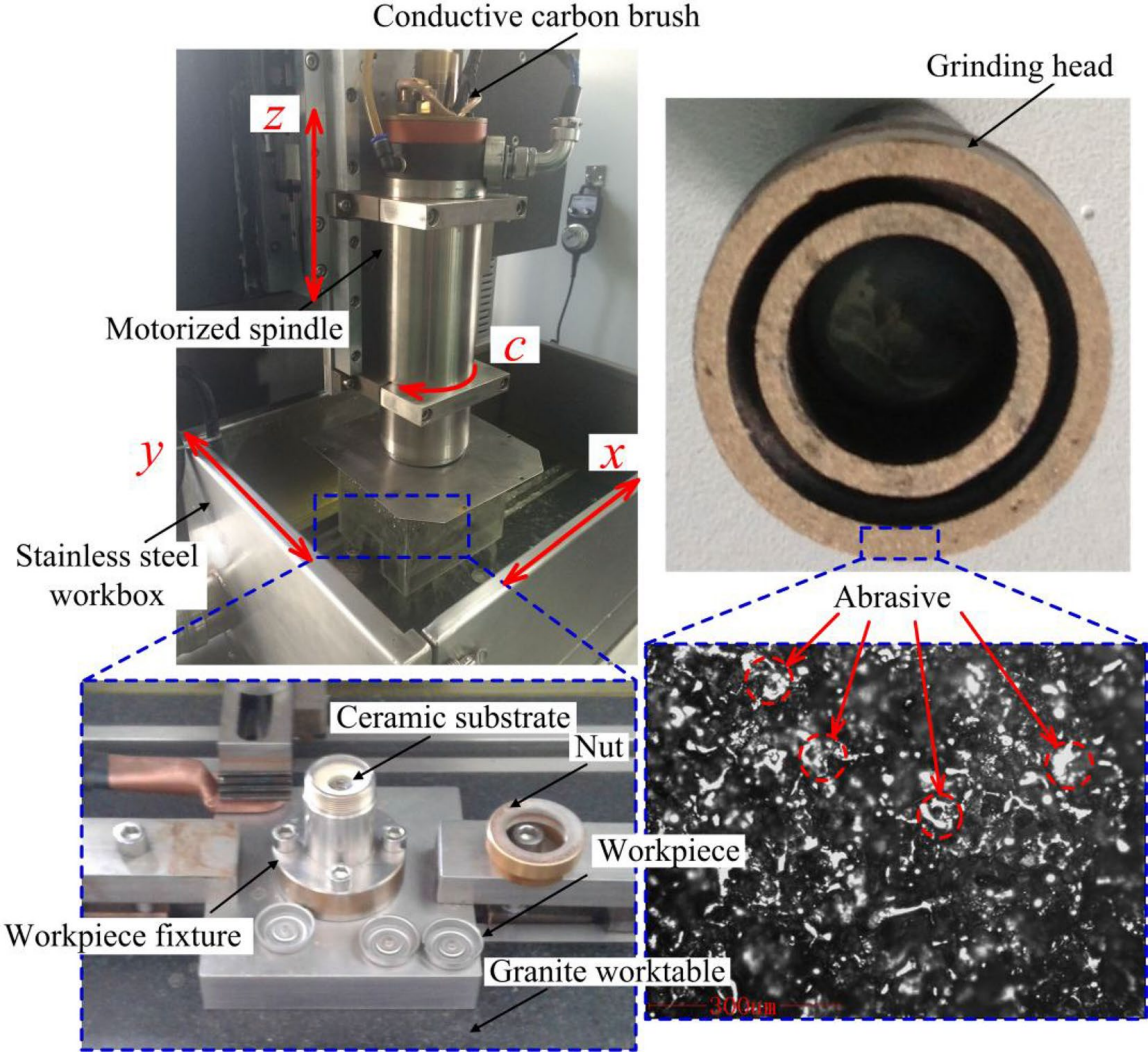
ECG system.

The ECG experiments of the torus surfaces of the stainless steel shaver cap were conducted based on the flow field simulation. Figure 5 shows the ECG system, which mainly includes an electric spindle, a marble platform, a workbox, a workpiece fixture, a rotating electrical conduction device, a rotating fluid-passing device, and an electrolyte circulating filtration system. The beryllium bronze-based diamond sintered grinding head is fixed at the bottom of the electric spindle through a chuck. The electric spindle drives the grinding head to rotate clockwise, and the frequency converter adjusts the rotation speed to be continuously adjustable in the range of 0 to 20,000 rpm. The electric carbon brush is installed on the top of the electric spindle, and is in flexible contact with the rotating mandrel inside the electric spindle to transmit the current. The rotating fluid-through joint is connected to the top of the mandrel and delivers high-velocity electrolytes to the hollow mandrel and the processing area. The workpiece is installed inside the fixture and pressed by a nut, and the workpiece’s bottom end is closely attached to the ceramic gasket. The workpiece material is SUS420J2. The double-ring thin-walled shaver cap is formed by high-speed stamping, and the hardness reaches 580 ± 20 HV after quenching and low-temperature annealing. The electrolyte is a sodium nitrate solution, and the electrolyte temperature is controlled at 25 ± 0.5 °C.

The effects of the rotating speed, feed rate, and the inlet pressure on the flatness and surface roughness of the torus surfaces were studied based on the machining parameters shown in Table 2. The flatness of the inner and outer torus surfaces, and the flatness of the double torus surfaces were inspected with the Hexagon imager, 60 sampling points were uniformly selected on the inner and outer torus surfaces respectively along the circumferential direction. With the aid of the Mahr roughness meter, the middle positions of the inner and outer torus surfaces were detected to determine the roughness values.

**Table 2:** Machining parameters of ECFG.

Machining parameter	Value
Abrasive particle size (μm)	74
Rotating speed *r* (rpm)	2400, 3000, 3600, 4200, 4800
Feed rate (mm/min)	0.2, 0.4, 0.6, 0.8, 1.0
Number of through-holes of grinding head	12
Through-hole diameter *d* (mm)	0.5
Applied voltage *U* (V)	10
Power duty cycle	50%
Power frequency (Hz)	1000
Inlet pressure *P*_in_ (MPa)	0.2, 0.25, 0.3, 0.35, 0.4
Electrolyte conductivity *κ* (S/m)	7.9

## Results and discussion

### Influence of grinding head rotating speed

**Figure 6:**
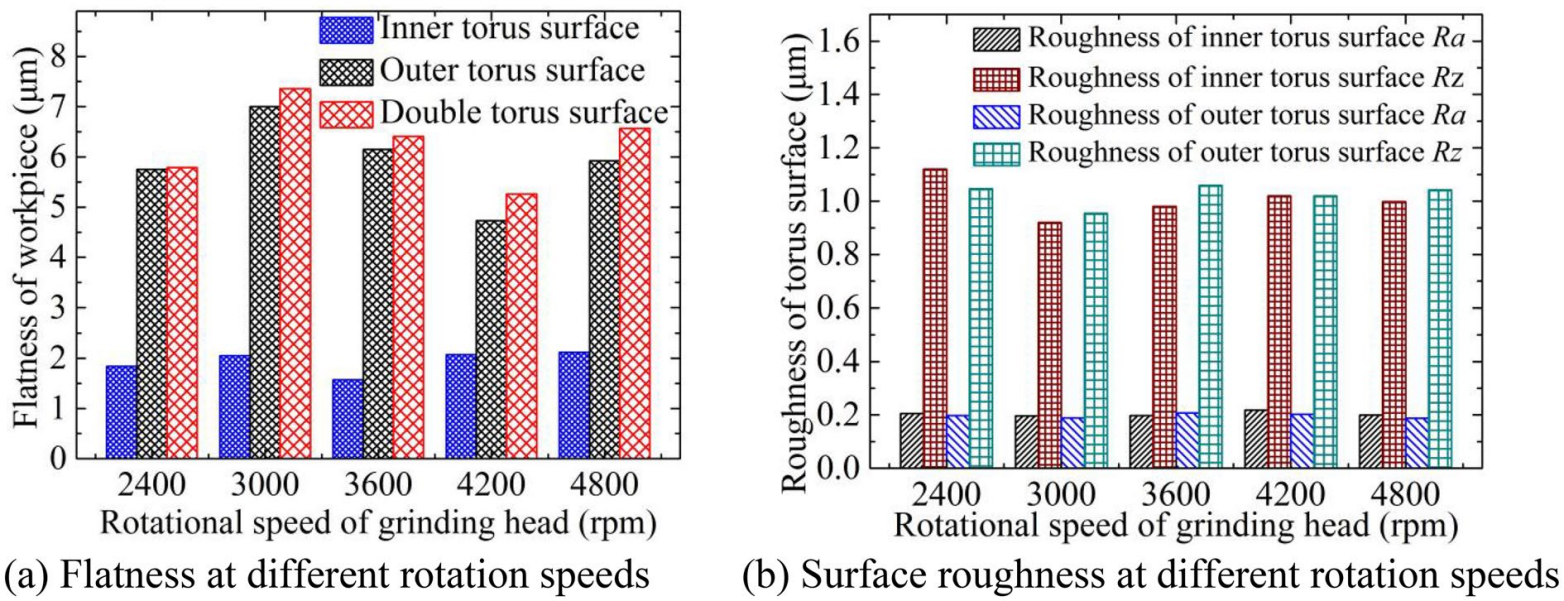
Flatness and roughness at different rotation speeds.

**Figure 7:**
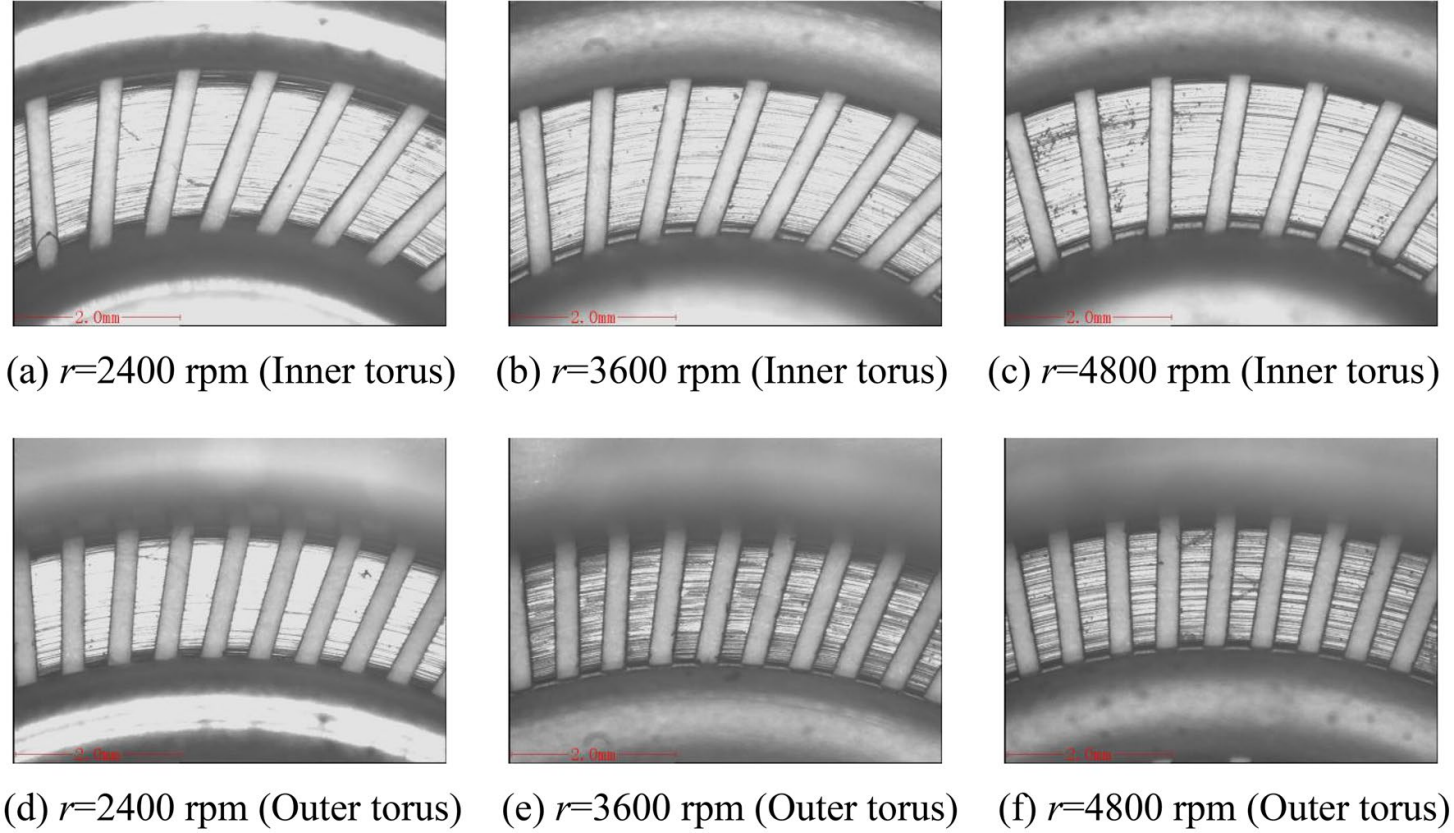
Inner and outer torus surfaces processed by different rotation speeds.

Figure 6a shows the change of flatness at different rotating speeds, and Fig. 6b depicts the change of surface roughness at different rotating speeds. The feed rate is 0.8 mm/min, and the inlet pressure is 0.3 MPa. The inner and outer torus surfaces processed by different rotating speeds are shown in Fig. 7, and the wall thickness of the torus is between 0.1 ± 0.003 mm through the detection of the Mitutoyo digital height gauge. The overall fluctuation of the flatness of the torus surfaces is smaller as the rotation speed increases, but the flatness of the inner torus surface is smaller than that of the outer torus surface. According to the simulation results of the through-hole structure, the difference in flow velocity between the inner and outer torus surfaces can be reduced by improving the diameter and distribution of the through-hole, thereby reducing the difference in the wall thickness of the torus. The electrolyte flow near the outer torus surface and the outlet is greatly affected by centrifugal force and Coriolis force. The intensified deflection of the flow direction causes non-uniform dissolution of the outer torus, thereby deteriorating the micro-leveling performance. However, the electrolyte flow in the inner torus surface is less affected by the rotation due to the low linear speed of the grinding head, and the matching effect of dissolution and grinding is better. Thus, the flatness of the inner torus surface is low.

The surface roughness *Ra* of the torus surfaces changes little with the increase of the rotating speed. When the rotation speed is between 2400 and 4800 rpm, the maximum height of the roughness profile *Rz* of the torus surfaces generally decreases first and then rises. However, *Rz* changes less when the rotation speed is higher than 3600 rpm. It can be seen from the simulation results that the flow velocity of the torus surfaces fluctuates slightly when the rotation speed is between 1800 and 6000 rpm, and the local dissolution rates of materials are relatively close. Therefore, the surface roughness *Ra* changes little with the increase of rotation speed. The number of abrasive grains passing through the machined surface per unit time is less when the rotating speed is low and the feed rate is high, and the workpiece surface is prone to produce significant microscopic protrusions and depressions. However, the grinding removal of passive film and workpiece substrate increases when the rotating speed is overly high, and the accumulation of products in the inter-electrode gap is not conducive to improving the uneven surface of the workpiece.

### Influence of inlet pressure

**Figure 8:**
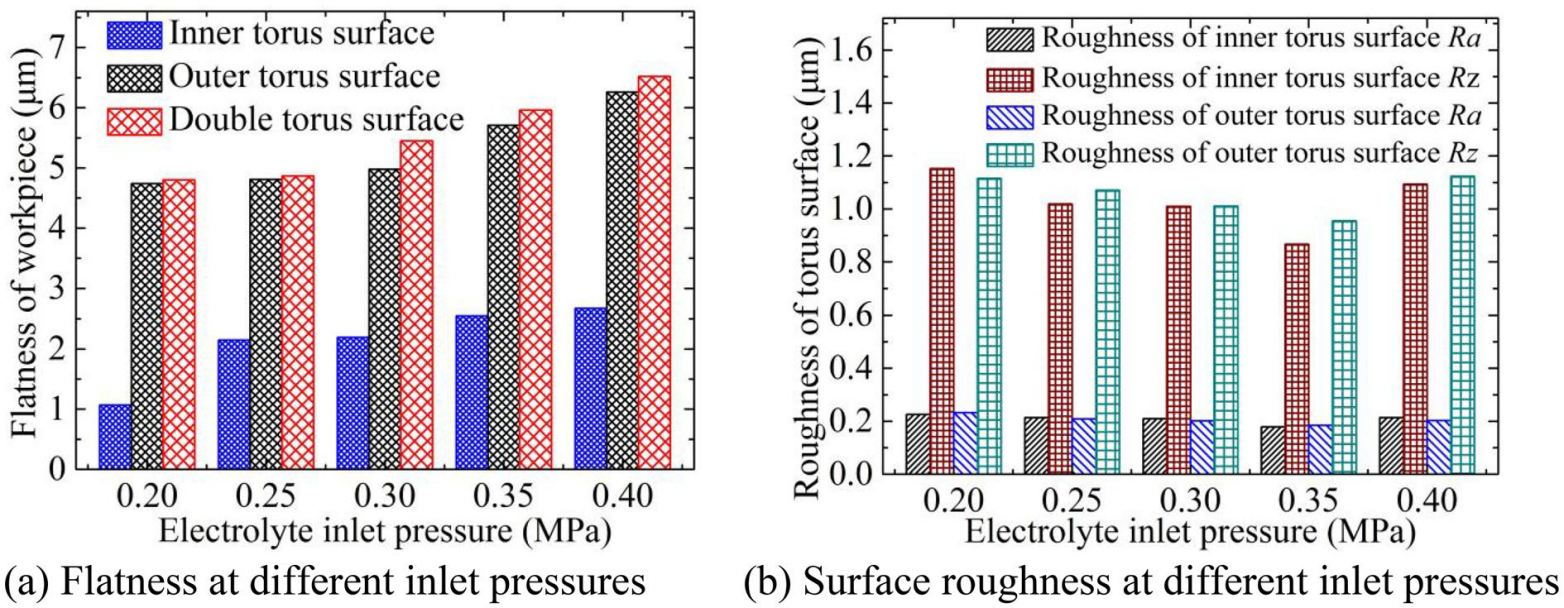
Flatness and roughness at different inlet pressures.

**Figure 9:**
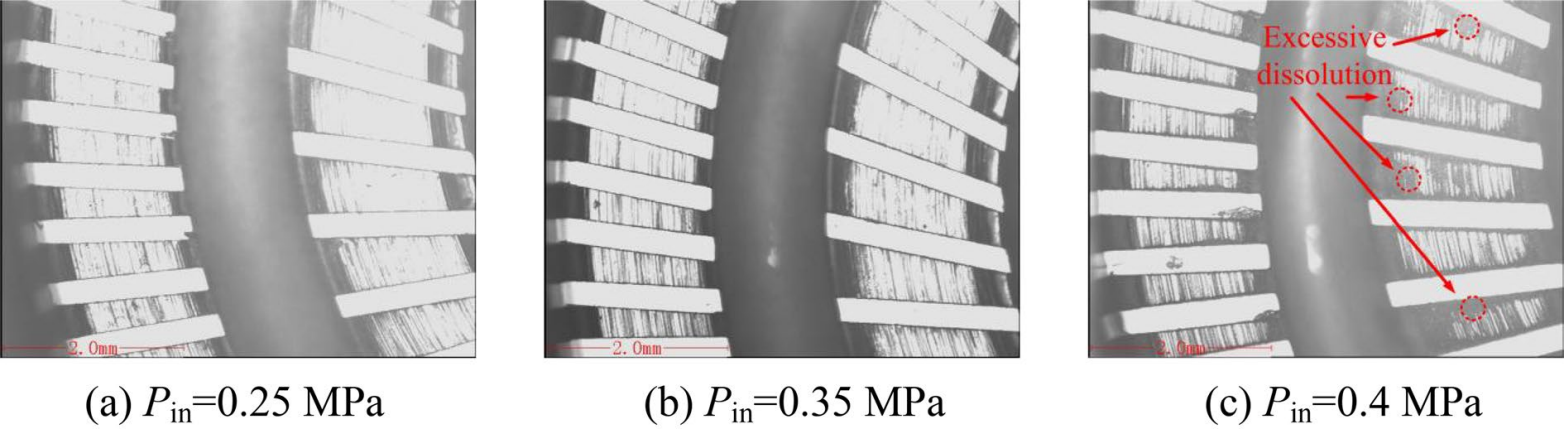
Torus surfaces processed by different inlet pressures.

Figure 8a shows the flatness of the torus surfaces at different inlet pressures, and Fig. 8b shows the variation of the surface roughness with the inlet pressure. The torus surfaces processed by different inlet pressures are shown in Fig. 9. The feed rate is 0.8 mm/min, and the rotation speed is 4200 rpm. The electrolyte flow in the inter-electrode gap increases significantly as the inlet pressure increases, which accelerates the electrochemical reaction on the workpiece surface, but the processing localization becomes worse. Therefore, the flatness of the torus surfaces increases with the increase of the inlet pressure. The surface roughness of the torus surfaces decreases first and then rises with the increase of the inlet pressure. The surface roughness *Ra* and the maximum height of the roughness profile *Rz* are both small when the inlet pressure is between 0.3 and 0.35 MPa. The electrolyte flow supply in the inter-electrode gap is insufficient and the abrasive particles are scratched on the workpiece surface when the inlet pressure is small, resulting in poor surface roughness. When the inlet pressure is high, the electrolyte flow supply in the inter-electrode gap is relatively sufficient, and the workpiece surface is prone to excessive dissolution and stray corrosion as shown in Fig. 9c. In addition, the strong dissolving effect increases the side reaction of materials, and the local material is even removed in the form of exfoliation, and the surface roughness has also deteriorated.

### Influence of feed rate

**Figure 10:**
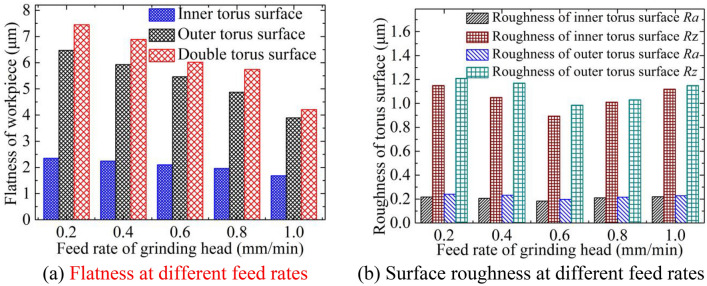
Flatness and roughness at different feed rates.

Figure 10a shows the flatness of the torus surfaces at different feed rates, and Fig. 10b depicts the surface roughness at different feed rates. The rotating speed is 4200 rpm, and the inlet pressure is 0.3 MPa. The flatness of the torus surfaces decreases significantly with the increase of the feed rate, while the surface roughness generally decreases first and then increases. When the feed rate is close to 0.8 mm/min, the flatness and surface roughness are smaller. The frontal gap between the grinding head and the workpiece bottom surface is large and the current density in the frontal gap is low when the feed rate is low, and the stainless steel is prone to selective dissolution, which is not conducive to the improvement of the leveling performance. The current density in the frontal gap increases as the feed rate increases, and the dissolution of the various phases in the stainless steel is relatively uniform. Thus, the matching effect of dissolution and grinding is also better. However, the scraping effect is significantly enhanced when the feed rate is overly high, and it is easy to leave deeper abrasive cutting marks on the workpiece surface.

### Fabrication of the torus surfaces of shaver cap

**Figure 11:**
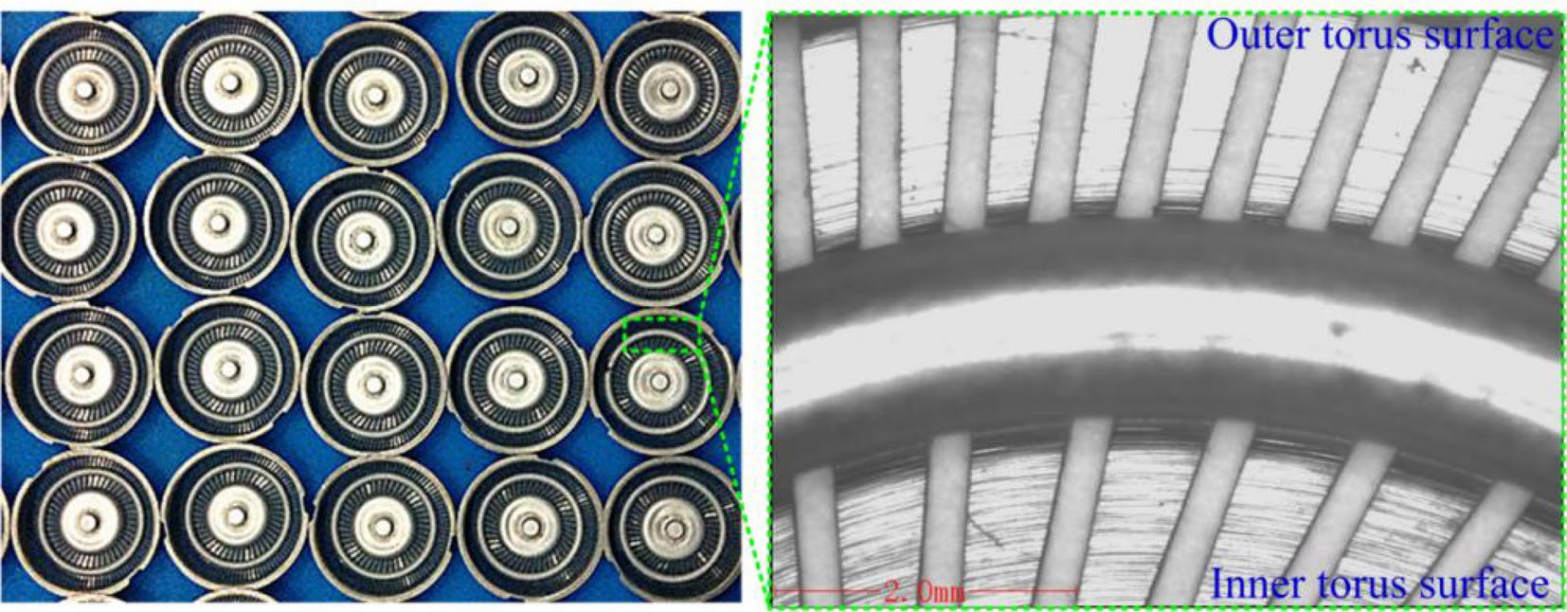
Torus surfaces processed with optimized parameters.

Based on the analysis of the influence of rotation speed, feed rate, and inlet pressure on the flatness and surface roughness of the torus surfaces, the parameter combination of an inlet pressure of 0.3 MPa, a rotation speed of 4200 rpm, and a feed rate of 0.8 mm/min was optimized for small-batch processing of stainless steel shaver cap. Figure 11 shows the torus surfaces of the shaver cap processed by ECG. The comprehensive flatness of the double torus surfaces was 5.7 μm, and the surface roughness *Ra* of the torus surfaces was close to 0.2 μm. During the machining process, the loss of the sintered grinding head was small, and the grinding head could realize the repeated machining of about 200 shaver caps after a one-time electric spark dressing.

## Conclusions

In this work, the effects of through-hole structure and rotation movement of grinding head on the flow field distribution were analyzed, and the effects of the rotation movement and the electrolyte parameters on the flatness and surface quality of the torus surfaces were experimentally investigated. In addition, the optimal matching of dissolution and grinding was promoted by optimizing the processing parameters, and the ECFG performance of high-hardness stainless steel was improved. The conclusions can be summarized as follows:

1. The flow field simulation results showed that the grinding head rotated at high speed to form a rotating flow, which was not conducive to the discharge of products in the inter-electrode gap. Moreover, the flow velocity difference between the inner and outer torus surfaces was still large, which was easy to produce uneven dissolution. The diameter and distribution of the through-holes had a significant effect on the flow velocity, and the difference in velocity could be reduced by reasonably designing the through-hole structure.
2. The experimental results showed that the use of a grinding head with through-holes on the sidewall could reduce the difference in the wall thickness of the shaver cap, while the rotation movement had little effect on the flatness and surface roughness of the torus surfaces. The high inlet pressure and low feed rate were easy to produce excessive dissolution, while the high feed rate and low inlet pressure were easy to generate deep cutting marks. In addition, the cutting marks could be reduced and the excessive dissolution could be eliminated by optimizing the machining parameters.
3. The high-quality processing of the high-hardness stainless steel shaver cap could be achieved by adopting the inner-jet ECG. The conductive grinding head loss was small, and the flatness and surface roughness of the repeatedly processed torus surfaces fluctuated little.
